# Application of deconvolutional networks for feature interpretability in epilepsy detection

**DOI:** 10.3389/fnins.2024.1539580

**Published:** 2025-01-24

**Authors:** Sihao Shao, Yu Zhou, Ruiheng Wu, Aiping Yang, Qiang Li

**Affiliations:** ^1^School of Microelectronics, Tianjin University, Tianjin, China; ^2^College of Medical Technology and Engineering, Henan University of Science and Technology, Luoyang, China; ^3^Department of Electronic and Electrical Engineering, Brunel University London, Uxbridge, United Kingdom; ^4^School of Electrical and Information Engineering, Tianjin University, Tianjin, China

**Keywords:** seizure detection, EEG, deconvolution network, interpretability analysis, deep learning

## Abstract

**Introduction:**

Scalp electroencephalography (EEG) is commonly used to assist in epilepsy detection. Even automated detection algorithms are already available to assist clinicians in reviewing EEG data, many algorithms used for seizure detection in epilepsy fail to account for the contributions of different channels. The Fully Convolutional Network (FCN) can provide the model’s interpretability but has not been applied in seizure detection.

**Methods:**

To address these challenges, a novel convolutional neural network (CNN) model, combining SE (Squeeze-and-Excitation) modules, was proposed on top of the FCN. The epilepsy detection performance for patient-independent was evaluated on the CHB-MIT dataset. Then, the SE module was removed from the model and integrated the model with Inception, ResNet, and CBAM modules separately.

**Results:**

The method showed superior advancement, stability, and reliability compared to the other three methods. The method demonstrated a G-Mean of 82.7% for sensitivity (SEN) and specificity (SPE) on the CHB-MIT dataset. In addition, The contributions of each channel to the seizure detection task have also been quantified, which led us to find that the FZ, CZ, PZ, FT9, FT10, and T8 brain regions have a more pronounced impact on epileptic seizures.

**Discussion:**

This article presents a novel algorithm for epilepsy detection that accurately identifies seizures in different patients and enhances the model’s interpretability.

## 1 Introduction

Epilepsy is a neurological disorder affecting the brain, with a lifetime prevalence of 7.6‰. It is characterized by recurrent and unprovoked epileptic seizures, making it a chronic condition (Fiest et al., 2017; Zhang et al., 2020). Based on the estimated figures by the World Health Organization, around 50 million individuals worldwide are impacted by epilepsy, which is categorized as a disorder affecting the central nervous system (Chakrabarti et al., 2021). Epileptic seizures, marked by spontaneous and irregular electrical activity in the brain, can cause profound and temporary changes in a person’s behavior, movement, sensory experience, and awareness of their surroundings (Nasiri and Clifford, 2021).

Identifying and treating epilepsy in its early stages can make a critical and valuable difference for individuals with this condition. Scalp electroencephalogram (EEG) is a non-intrusive technique for measuring the electrical activity in the brain and is a widely employed supplementary examination in diagnosing epilepsy (Liang et al., 2020). During a seizure episode, the patient’s EEG will exhibit significant abnormal patterns (Staba et al., 2014). Doctors can use the examination of an EEG to help determine if epilepsy is occurring. However, reviewing long-term EEG requires doctors to invest much time and effort. Therefore, developing automated epilepsy detection algorithms is crucial (Si et al., 2023).

Researchers are actively working toward the development of automated detection of epileptic seizures using EEG data. From the initial attempts using hardware circuits to later utilizing time-domain information and threshold-based methods for seizure detection. The subsequent development involves using frequency-domain features and extracting time-frequency characteristics (Xia et al., 2015) for seizure detection.

Deep learning models are more resilient in computer vision tasks compared to features extracted manually since their introduction (Chen et al., 2024), speech recognition (Eris and Akbal, 2024), and natural language processing (Luo et al., 2024). Therefore, utilizing deep learning techniques to detect epileptic seizures using EEG signals automatically has shown significant prospects in making the most suitable and fastest clinical decisions (Ahmad et al., 2023). In the last few years, various deep learning models have been utilized for epileptic seizure detection, including recurrent neural networks (Tuncer and Bolat, 2022), generative adversarial networks (Rasheed et al., 2021), deep neural networks (Liu and Richardson, 2021), hierarchical neural networks (Hu et al., 2021), and convolutional neural networks. These models have achieved promising results (Kaur et al., 2022).

Convolutional networks have shown further improvements in performance after being trained end-to-end, pixel by pixel. With the introduction of a Fully Convolutional Network (FCN), the neural network design could handle inputs of varying sizes and produce correspondingly-sized outputs through highly effective inference and learning mechanisms (Chou et al., 2023). However, FCN has not yet been widely applied in seizure detection. Meanwhile, previous deep learning algorithms often neglected the contributions of different channels to the classification task, resulting in models with limited interpretability.

To overcome the problem above, an independent epilepsy detection algorithm based on deep learning was introduced. algorithm can autonomously extract temporal and spatial information from multi-channel EEG data, enabling precise identification of seizure events across diverse patients. This paper makes several key contributions, including:

λ A CNN model detection algorithm incorporating the SE (Squeeze-and-Excitation) module was proposed. This method has been evaluated on the CHB-MIT dataset and has achieved excellent performance.

λ For the first time, the upsampling method in the FCN model was applied to seizure detection, achieved through the utilization of deconvolution, which upscales the downscaled images from the convolutional layers back to their original size, allowing for the visualization of the processed EEG.

λ The EEG data was subjected to weight normalization to quantify the varying contributions of each channel to seizure detection. Then, the brain regions FZ, CZ, PZ, FT9, FT10, and T8 were explored for their more significant influence on epileptogenesis. As a result, the interpretability of the model was enhanced.

The remaining section are as follows. Section “2 Deep learning” analyzes related methods for epilepsy detection using deep learning, including CNNs, LSTM and Transformer networks. Subsequently, FCN was introduced. Section “3 Materials and methods” outlines the approach employed in this study, encompassing the dataset, enhanced seizure detection models, and the training methodology. Section “4 Results” showcases the experimental results, featuring a comparison with the baseline model and a visual examination of the EEG channel weights. Section “5 Discussion” evaluates the paper by examining its performance and the interpretability of the proposed model. Section “6 Conclusion” provides a comprehensive summary of the research in this paper, presents the obtained results, and discusses the existing limitations and future prospects.

## 2 Related work

### 2.1 Deep learning

With the advancement of theoretical principles of computer science and the availability of computational resources, deep learning has outperformed conventional machine learning and achieved better results in many fields (Si et al., 2021). Deep learning algorithms can extract data features that are automated and robust without the biases and complexities involved in feature engineering by hand (LeCun et al., 2015). As a result, some deep learning methods have been used for detecting epileptic seizures, including CNN (Du and Liu, 2022; O’Shea et al., 2020; Abdulwahhab et al., 2024),Long Short-Term Memory (LSTM) (Alharbi et al., 2024; Singh and Malhotra, 2022), Transformer models (Hu et al., 2023), and so on.

Ozdemir et al. (2021) introduced a novel approach that utilizes the Short-Time Fourier Transform (SST) and CNN to detect and forecast epileptic seizure episodes. The proposed method was evaluated on the IKCU and CHB-MIT datasets and achieved high accuracy and precision in detecting seizure episodes based on segments. Albaqami et al. (2023) proposed a novel deep learning network called Multi-Path Seizure Classification Network (MP-SeizNet), which consists of a CNN and a Bidirectional Long Short-Term Memory Neural Network (Bi-LSTM). The proposed method was evaluated on the EEG epilepsy database from the Temple University Hospital and achieved an F1 score of 87.6%. Li suggested a method for predicting seizures in EEG data, which involves using a Transformer-guided CNN (TGCNN) to extract both local and global characteristics efficiently (Li et al., 2022). This method combined different benefits of both CNN and Transformer models. It achieved a sensitivity of 82.2%, an FPR (False Positive Rate) of 0.06/h, and an AUC (Area Under the Curve) of 83.5% on the Kaggle database.

Since Shelhamer et al. (2017) proposed the FCN, it has found diverse applications across multiple domains, such as image segmentation and hyperspectral image classification, achieving excellent results. Jiang introduced a new end-to-end, pixel-level Fully Convolutional Spatial Propagation Network (FCSPN), consisting of a 3D-fully convolutional network (3D-FCN) and a convolutional spatial propagation network (CSPN) (Jiang et al., 2021). This method has achieved advanced performance in hyperspectral image (HSI) classification. Shen et al. (2021) proposed a method that combines a deep FCN with an effective non-local module (ENL-FCN) for hyperspectral HSI classification. The FCN takes the entire HSI as input in the proposed framework and extracts spectral-spatial information within local receptive fields (Shen et al., 2021). The proposed method achieved excellent classification performance while maintaining lower computational costs.

### 2.2 Explainable deep learning architecture

In recent years, FCN has also been applied in medical image processing. For example, Qiu has suggested a hybrid framework that merges FCN and Multi-Layer Perceptron (MLP) to generate high-resolution probability maps of diseases using local brain structures. And generate precise and intuitive individual Alzheimer’s disease risk visualization maps to achieve accurate diagnosis. The diagnostic accuracy achieved by this framework is comparable to that of neurologists (Qiu et al., 2020).

However, FCN has not yet been applied in seizure detection. Meanwhile, Due to the opaque nature of deep learning models, interpreting and comprehending the learned models in an intuitive manner remains a significant challenge (Khan et al., 2024). In particular, different channels of EEG signals contribute differently to seizure detection and the impact of different brain regions on seizure occurrence in the brain. Therefore, a CNN detection algorithm that combines an SE module on top of the FCN was proposed. The SE module offers the advantage of being easily pluggable and has not been previously applied to EEG-based seizure detection algorithms using deep learning. The algorithm not only performs epilepsy detection but also obtains the weights of each EEG signal channel, after which the corresponding brain regions can be found according to the cascade system correspondence. It enhances the model’s performance and interpretability.

## 3 Materials and methods

### 3.1 EEG data

The CHB-MIT database was utilized as the EEG database in this research. The details are as follows (Table 1). Records from 22 patients were available for this study, including 5 boys aged between 3 and 22 and 17 girls aged between 1.5 and 19. These records were divided into 23 individual cases, with one (chb21) belonging to the same patient as the case chb01 but collected 1.5 years later. The specific details of each subject can be found in Table 2. The chb24 case was included in the dataset in December 2010. However, the exact age and gender of this individual are not accurate. Each patient has between 9 and 42 consecutive.edf files, with the majority of these files containing precise one-hour signals. Each patient has an average of 5.75 seizure episodes of varying durations. In this database, the EEG signals were universally sampled at 256 Hz with a 16-bit resolution. Most EEG recordings include 18–23 bipolar EEG electrodes arranged according to the international 10–20 system (Abdallah et al., 2023; Liu et al., 2022).

**TABLE 1:** CHB-MIT dataset details.

Dataset	CHB-MIT
Type	Scalp EEG
Subjects	23
Male	5
Female	17
Age	Between 1.5 and 22
Electrodes	23
Sampling rate	256 Hz
EDF file for each patient	Between 9 and 42
Average number of seizures per Patient	5.75
Electrode positions	The international 10–20 method

**TABLE 2:** Specifics of each subject.

No.	Sex	Age	Number of used seizures	Total duration (h)	Mean seizure duration(s)
1	F	11	7	40.55	63.15
2	M	11	3	35.27	57.34
3	F	14	7	38.00	57.43
4	M	22	4	156.07	94.50
5	F	7	5	39.00	111.60
6	F	1.5	10	66.74	15.30
7	F	14.5	3	67.05	108.34
8	M	3.5	5	20.01	183.80
9	F	10	4	67.87	69.00
10	M	3	6	50.02	65.50
11	F	12	3	34.79	268.67
12	F	2	27	20.69	36.63
13	F	3	12	33.00	44.59
14	F	9	8	26.00	22.13
15	M	16	20	40.01	99.60
16	F	7	10	19.00	8.40
17	F	12	3	21.01	97.67
18	F	18	6	35.63	52.84
19	F	19	3	29.93	78.67
20	F	6	8	27.60	36.75
21	F	13	4	32.83	49.75
22	F	9	3	31.00	68.00
23	F	6	7	26.56	60.58
24	N/A	N/A	16	21.30	31.94
Summary	–	9.98	184	979.93	74.22

### 3.2 Data preprocessing

The processed data was passed through a sixth-order Butterworth bandpass filter from 1 to 60 Hz to eliminate baseline noise and high-frequency interference. The raw EEG signals were resampled to a frequency of 128 Hz to reduce the data in the deep learning model. Because epileptic seizures have an intermittent nature, during seizures, EEG recordings are usually of shorter duration compared to non-seizure periods. Therefore, following previous research, the filtered EEG data was segmented into 2-s epochs using a sliding window analysis (Park et al., 2020). All the operations above were performed using the MNE-Python library in Python (Jaiswal et al., 2020).

### 3.3 Proposed method

**FIGURE 1:**
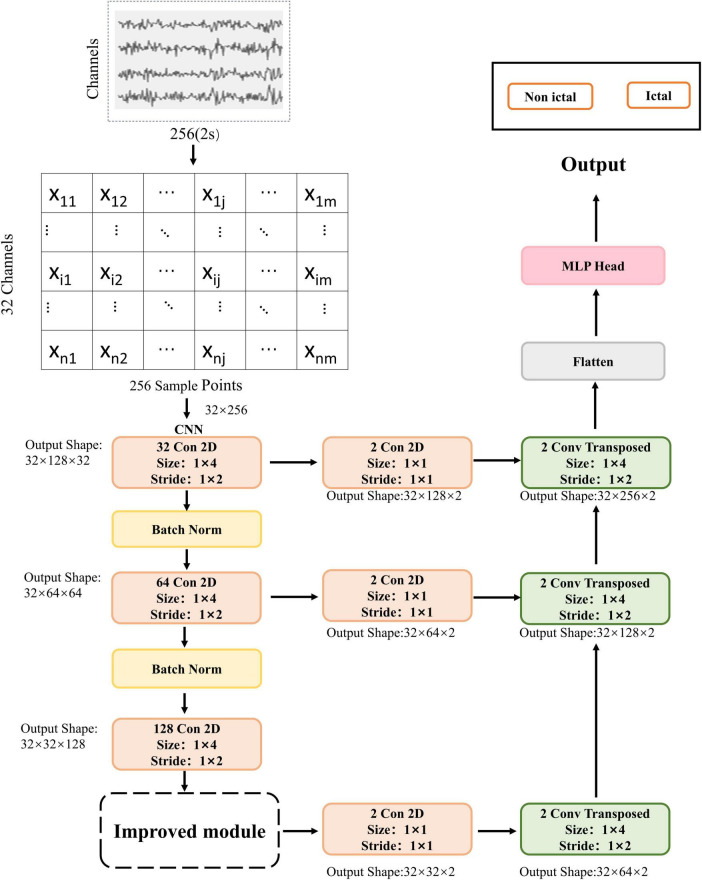
The architecture and flow diagram of the proposed method. Conv Transposed: deconvolution.

A lightweight CNN combined with an SE module was used to build a seizure detection algorithm. Figure 1 shows a schematic representation of the proposed method’s framework, which draws inspiration from the FCN architecture for the model design, mainly utilizing its skip connections and deconvolution (upsampling) approach.

The EEG segment was initially configured with 32 channels. Due to each component containing 2 s of EEG data with a sampling rate of 128 Hz, the shape of the input EEG segment would be (32, 256). The data segment is passed through a two-dimensional convolutional layer in the next step. This layer consists of 32 filters with a kernel size of (1, 4) and a stride of (1, 2). Batch normalization and ReLU activation extract low-level features and minimize data superfluity. Following that, the data is passed through a convolutional layer with 64 filters, a kernel size of (1, 4), and a stride of (1, 2). Batch normalization and ReLU activation are applied again. The core idea of batch normalization is to perform standardization on the inputs of each layer in the network. Batch normalization layers can maintain the stability of the input data distribution while preserving the network’s nonlinear expressive power. The data is then passed through another convolutional layer with 128 filters, a kernel size of (1, 4), and a stride of (1, 2). At this stage, the output feature map shape would be (32 × 32 × 128). The output features are passed through the SE module, which focuses on channel relationships. The goal of the SE module is to enable the model to learn the relative significance of diverse channel features automatically (Hu et al., 2020).

The feature maps are then passed through a convolutional layer with two filters, a kernel size of (1, 1) and a stride of (1, 1). Next, they enter the first deconvolutional layer with two filters, a kernel size of (1, 4) and a stride of (1, 2). At this stage, the output feature map shape would be (32 × 64 × 2). The second convolutional layer has two filters, a kernel size of (1, 1) and a stride of (1, 1). The output features from the first convolutional layer are simultaneously fed into a deconvolutional layer with two filters, a kernel size of (1, 4), and a stride of (1, 2). At this stage, the output feature map shape would be (32 × 128 × 2). The output features from the first convolutional layer are passed through a convolutional layer with two filters, a kernel size of (1, 1), and a stride of (1, 1). Simultaneously, the second deconvolutional layer’s output features are fed into a deconvolutional layer with two filters, a kernel size of (1, 4), and a stride of (1, 2). At this stage, the output feature map shape would be (32 × 256 × 2). Finally, the output features are passed through a flattened layer and a Multilayer Perceptron (MLP) to output probabilities of epileptic and non-epileptic seizures (Table 3).

**TABLE 3:** The number of parameters in each layer of the network.

Layer (type)	Output shape
Input	32 × 256
CNN_1	32 × 128 × 32
Batch_Normalization_1	32 × 128 × 32
CNN_2	32 × 64 × 64
Batch_Normalization_2	32 × 64 × 64
CNN_3	32 × 32 × 128
SE module	32 × 32 × 128
CNN_4	32 × 32 × 2
CNN_Transposed_1	32 × 64 × 2
CNN_Transposed_2	32 × 128 × 2
CNN_Transposed_3	32 × 256 × 2
CNN_1_1	32 × 128 × 2
CNN_2_1	32 × 64 × 2
Flatten	16,384
MLP head	2

**FIGURE 2:**
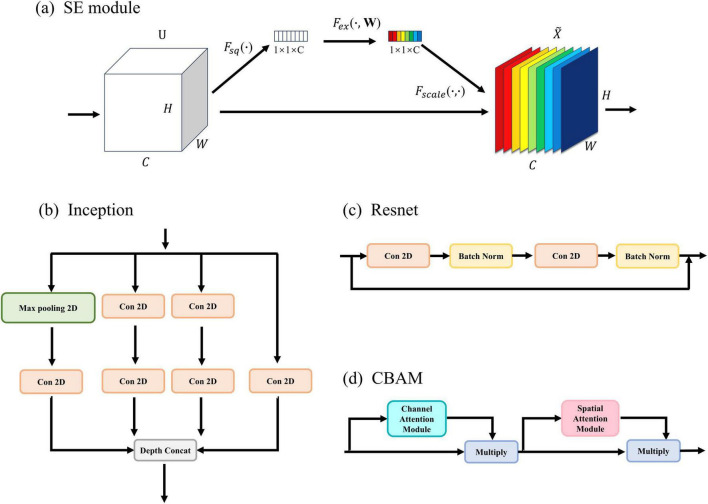
Improved module: **(A)** the detailed architecture of SE (Squeeze and Excitation). **(B)** the detailed architecture of Inception. **(C)** the detailed architecture of Resnet. **(D)** the detailed architecture of CBAM.


The details of SE are shown in Figure 2A. In SE, the first step is to perform a Squeeze operation (*F*_*sq*_(⋅)) on the input feature U, resulting in *z_c_*. This operation (1) decreases the number of dimensions in the feature map by taking the average value and extracting global feature information.

(1) $$z_{c}=F_{s\,q}\,\left(u_{c}\right)=\frac{1}{H\times W}\,\sum_{i=1}^{H}\sum_{j=1}^{W}u_{c}\,\left(i,j\right)$$

Next, the Excitation operation (*F*_*ex*_(⋅)) is performed, where z represents the previous step’s output, and W1 and W2 denote linear layers. The computed s (2) in this step is the module’s core, meaning the weights for each channel.


(2) $$s=F_{e\,x}\,\left(z,W\right)=\sigma \,\left(g\,\left(z,W\right)\right)=\sigma \,\left(W_{2}\,\delta \,\left(W_{1}\,z\right)\right)$$

Finally, the Scale operation [*F*_*scale*_(⋅)] is performed. This results in the final output $\overset{ˇ}{X}$ of the SE module.


(3) $$\overset{ˇ}{X}=F_{s\,c\,a\,l\,e}\,\left(u_{c},s_{c}\right)=s_{c}$$

Where *u_c_* represents a channel in U, and *s_c_* means the weight of the channel. Therefore, it is equivalent to multiplying the value of each channel by its weight.

### 3.4 Training and testing strategy

A leave-one-patient-out cross-validation approach was implemented to assess the effectiveness of the method, specifically designed for medical utilization. In the dataset of N patients, one patient’s data served as the test set, while the data from the remaining N-1 patients were employed as the training and validation sets. Each patient’s data was sequentially utilized as the test set, repeating this process N times to ensure comprehensive coverage (Park et al., 2020). Within the data of N-1 patients, 20% was arbitrarily picked as the validation set, while the remaining 80% was utilized for training the model.

**FIGURE 3:**
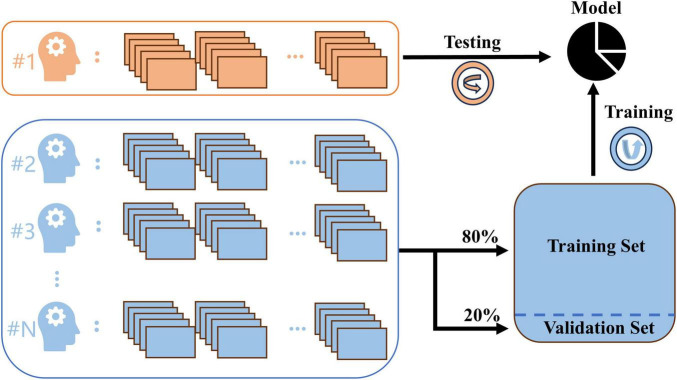
The diagram of leave-one-patient-out cross-validation.

The specifics of the training and testing approach are illustrated in Figure 3. During the training process, a batch size of 32 was utilized, and the model was trained for 100 epochs. The model that achieved the highest validation accuracy was chosen for the testing stage. The model was developed using Python 3.10 and Keras 2.4.3, and it is configured to operate on an NVIDIA GeForce RTX 3080 Ti GPU.

## 4 Results

Several metrics were presented for each technique to thoroughly and fairly assess their results. These metrics include sensitivity (SEN), specificity (SPE), geometric mean (G-Mean), and the area under the receiver operating curve (AUC). Sensitivity refers to the proportion of all actual epileptic samples that are correctly classified as epileptic. High sensitivity means that the model is capable of accurately identifying most epileptic seizures. Specificity refers to the proportion of all actual non-epileptic samples that are correctly classified as non-epileptic. High specificity means that the model is capable of accurately identifying most non-epileptic situations. The geometric mean is the geometric mean of sensitivity and specificity, used to balance the model’s sensitivity and specificity. A high AUC value indicates that the model has a high level of accuracy in distinguishing between epileptic and non-epileptic samples. The expressions for SEN, SPE, ACC, and G-Mean are as follows:


(4) $$\text{S}\,\mathrm{EN}=\frac{\text{TP}}{\mathrm{TP}+\mathrm{FN}}$$

(5) $$\mathrm{SPE}=\frac{\text{TN}}{\mathrm{TN}+\mathrm{FP}}$$

(6) $$\text{G}-\mathrm{Mean}=\sqrt{\mathrm{SEN}\times \mathrm{SPE}}$$

(7) $$\mathrm{Accuracy}=\frac{\mathrm{TP}+\mathrm{TN}}{\mathrm{TP}+\mathrm{TN}+\mathrm{FP}+\mathrm{FN}}$$

FP, TP, FN, and TN denote false positive, true positive, false negative, and true negative.

(1) Evaluation and comparison

Table 4 summarizes the detection evaluation results of the SE model on the CHB-MIT dataset. For instance, the results obtained from chb02 indicate that the model was trained using data from 22 patients, excluding chb02, and chb02’s data was used solely for testing the model’s performance. The SE model achieved 79.6% SEN, 86.0% SPE, 82.7% G-Mean, and 0.89 AUC.

**TABLE 4:** The performance of the SE model on the CHB-MIT dataset.

ID	Sen (%)	Spe (%)	G-Mean (%)	Acc	Auc
chb01	96.8%	93.6%	95.2%	0.94	0.99
chb02	80.8%	92.9%	86.7%	0.93	0.96
chb03	99.5%	79.9%	89.2%	0.90	0.99
chb04	77.2%	88.0%	82.4%	0.88	0.89
chb05	96.1%	82.7%	89.1%	0.83	0.98
chb06	56.5%	75.7%	65.4%	0.75	0.51
chb07	92.6%	87.8%	90.2%	0.88	0.94
chb08	64.5%	92.8%	77.4%	0.92	0.86
chb09	99.3%	61.3%	78.0%	0.62	0.99
chb10	95.7%	77.6%	86.2%	0.78	0.97
chb11	56.9%	96.0%	73.9%	0.95	0.82
chb12	53.6%	86.0%	67.9%	0.85	0.89
chb13	57.4%	92.3%	72.8%	0.91	0.80
chb14	55.7%	87.4%	69.8%	0.87	0.73
chb15	78.7%	94.2%	86.1%	0.92	0.88
chb16	66.0%	84.6%	74.7%	0.85	0.79
chb17	94.2%	82.3%	88.0%	0.82	0.96
chb18	72.0%	79.5%	75.7%	0.79	0.88
chb19	81.9%	88.1%	84.9%	0.88	0.93
chb20	76.2%	79.4%	77.8%	0.90	0.84
chb21	82.4%	89.7%	86.0%	0.90	0.94
chb22	98.5%	95.4%	97.0%	0.95	1.00
chb23	97.2%	91.7%	94.4%	0.92	0.98
(Mean ± SEM)	79.6% ± 0.91%	86.0% ± 0.57%	82.7% ± 0.23%	0.87 ± 0.009	0.89 ± 0.006

SEM: standard error of mean.

To further showcase the efficacy of the FCN architecture, three new networks were constructed by removing the SE module from the proposed network and incorporating Inception (Figure 2B), Resnet (Figure 2C), and CBAM (Figure 2D) modules. Please refer to the Supplementary material for more details. In addition, to demonstrate the excellence of the approach, it is crucial to compare it with other excellent seizure detection methods, including EEGNet (Lawhern et al., 2018), CNN+LSTM (Xu et al., 2020), and Wei-CNN (Craley et al., 2021). Table 5 provides a summary of the result comparison of each technique on the CHB-MIT datasets.

**TABLE 5:** G-mean, Acc, Auc and Lat of each method.

Method	G-mean (%)	Acc	Auc	Lat(s)
EEGNet	81.3	0.84	0.85	13.9
CNN+LSTM	81.2	0.84	0.83	16.2
Wei-CNN	79.7	0.83	0.77	18.7
SE model	82.7	0.87	0.89	6.1
Inception	83.4	0.87	0.88	8.9
ResNet	84.6	0.83	0.87	7.3
CBAM	84.3	0.85	0.84	7.0

Lat: latency.

**FIGURE 4:**
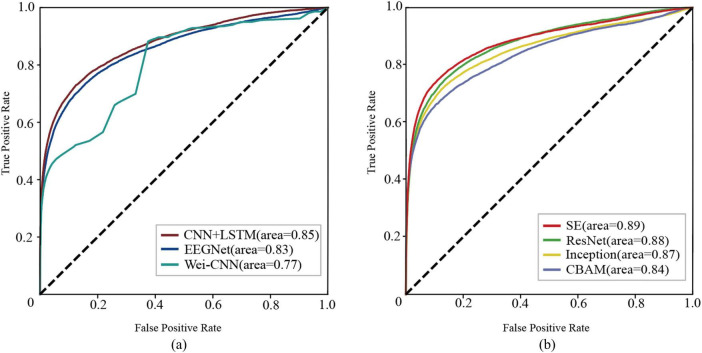
The receiver operating characteristic curves (ROC) illustrate the performance of each network. **(A)** baseline methods **(B)** the proposed methods.

**FIGURE 5:**
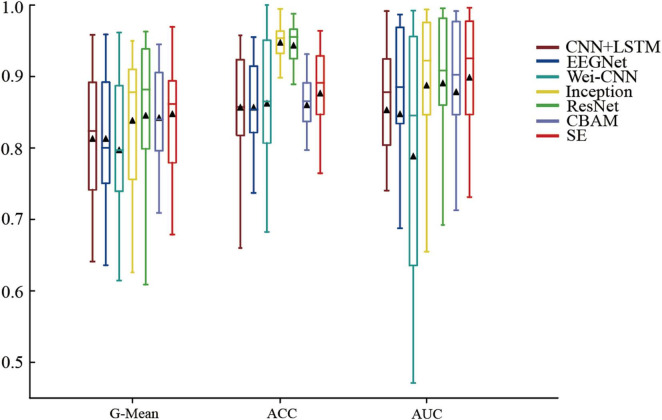
Box plot of the methods’ and baseline methods’ performance on the CHB-MIT dataset.

The methods mentioned above were experimented on the CHBMIT dataset, and ROC curves (Figure 4) and performance boxplots (Figure 5) were generated to compare the proposed methods with the baseline methods. It can be observed that the method achieves higher G-Mean, ACC, and AUC, indicating superior performance.

To evaluate the contribution of the SE module to the performance improvement of the proposed epilepsy detection model, an ablation experiment was conducted on the proposed method. Table 6 summarizes the results of the ablation experiments conducted on the CHB-MIT dataset. The results show that the SE module enhances the model’s performance across all five evaluation metrics. Compared to other metrics, the SE module demonstrates the greatest improvement in the model’s Spe.

**TABLE 6:** The results of the ablation experiments on the CHB-MIT dataset.

Dataset	SE module	Sen (%)	Spe (%)	G-Mean (%)	Acc	Auc
CHB-MIT	×	76.2%	81.8%	79.0%	0.84	0.85
	√	79.6%	**86.0%**	**82.7%**	**0.87**	**0.89**

Bold values means effective.

(2) Explainability analysis

The main innovation of the proposed method is inspired by the FCN architecture, specifically utilizing skip connections and deconvolution (upsampling) techniques. The size of feature maps decreases after the preceding convolutional operations, and the deconvolution restores the reduced feature maps to their original size. After the final deconvolution, the original feature map was extracted. This layer was removed and processed into a 2560 × 32 feature matrix in the main program. Since the sampling frequency is 128, 2560 represents a 20-s EEG feature map, and 32 represents 32 channels, including zero-padding channels.

Perform the following operations on the feature matrix:


(8) $$\text{A}=\left[{\text{a}}_{1,1},{\text{a}}_{2,1}\,{\text{a}}_{n,1}\right]=\text{Max}\,\left(\left|{\text{b}}_{1,1}\right|,\left|{\text{b}}_{1,2}\right|\,\ldots \,\left|{\text{b}}_{1,t}\right|\right),$$

$$\text{Max}\,\left(\left|{\text{b}}_{2,1}\right|,\left|{\text{b}}_{2,2}\right|\,\ldots \,\left|{\text{b}}_{2,t}\right|\right)$$

$$\ldots \text{Max}\left(|{\text{b}}_{n,1}|,|{\text{b}}_{n,2}|\ldots |{\text{b}}_{n,t}|\right)]$$

(9) $$C=[\left(\frac{\left|b_{1,1}\right|}{a_{1,1}},\frac{\left|b_{1,2}\right|}{a_{1,1}}\ldots \frac{\left|b_{1,t}\right|}{a_{1,1}}\right),$$

$$\left(\frac{\left|b_{2,1}\right|}{a_{2,1}},\frac{\left|b_{2,2}\right|}{a_{2,1}}\,\ldots \,\frac{\left|b_{2,t}\right|}{a_{2,1}}\right)$$

$$\ldots \left(\frac{\left|b_{n,1}\right|}{a_{n,1}},\frac{\left|b_{n,2}\right|}{a_{n,1}}\ldots \frac{\left|b_{n,t}\right|}{a_{n,1}}\right)]$$

Where matrix b ∈ R^(n × t). Took the absolute value of each value in the feature matrix and normalized each row by dividing it by the maximum value of that row. Mapped the data of each pixel to the range of 0 to 1, resulting in the weight matrix.

**FIGURE 6:**
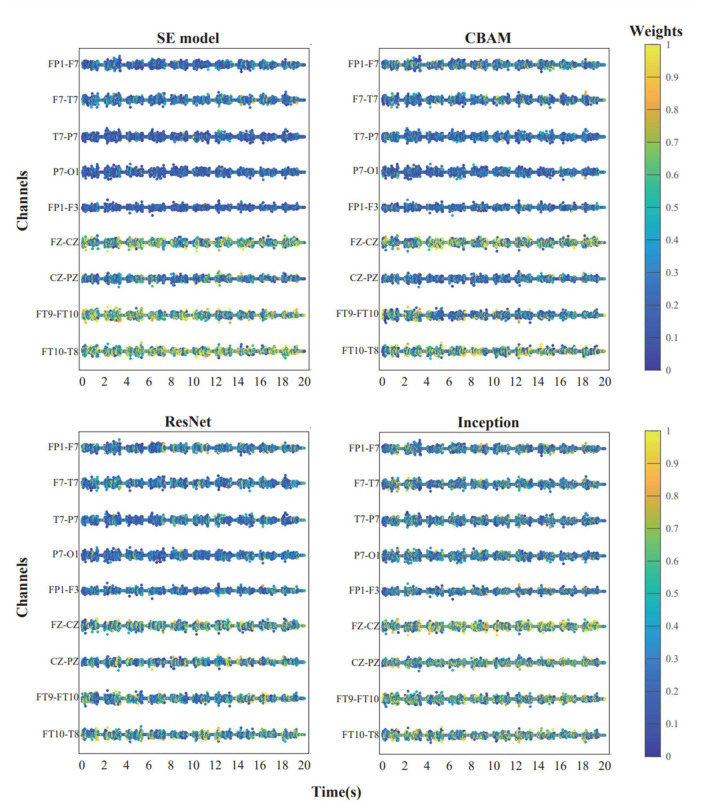
The weight distribution of each channel in each model during the first 20 s before the initial seizure in patient chb04. The channel weights corresponding to the brain regions FZ-CZ, FT9-FT10, FT10-T8, and CZ-PZ are comparatively higher.

**FIGURE 7:**
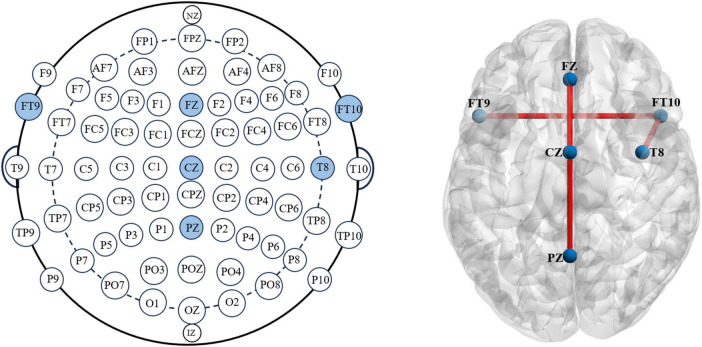
According to the international 10–20 electrode placement system, the brain regions FZ, CZ, PZ, FT9, FT10, and T8 are marked. The BrainViewer is used to visualize the brain atlas, labeling the corresponding areas and establishing functional connections at those positions.

The diagram below shows the weights of each channel in the model during the first seizure of patient CHB04 (Figure 6). The weight matrix was visualized by naming the rows of channels as the names of the corresponding brain regions. The channels corresponding to FP1-F7, F7-T7, T7-P7, P7-O1, FP1-F3, FZ-CZ, CZ-PZ, FT9-FT10, and FT10-T8 were filtered out. It can be observed that during the first 20 s of the seizure, the brain regions FZ-CZ, FT9-FT10, and FT10-T8 have a significant impact on the onset of epilepsy. For the weight diagrams of the Inception, Resnet, and CBAM modules (Figure 6), in addition to observing that the channels corresponding to the brain regions FZ-CZ, FT9-FT10, and FT10-T8 have relatively large weights, the channels corresponding to the CZ-PZ brain region have significant weights. A brain region map can be generated based on the channel diagram and mark the corresponding brain regions (Figure 7). Indeed, this further enhanced the interpretability of the FCN structure.

## 5 Discussion

### 5.1 CNN algorithm and model benefits

The EEG is an often utilized medical technique for diagnosing epilepsy (Duan et al., 2022). In the past few years, extensive interest has been in seizure detection based on EEG. EEGNet, proposed by Lawhern, is a compact CNN used for brain-computer interface based on EEG (Lawhern et al., 2018). Xu et al. (2020) proposed a 1D CNN-LSTM model for automatically identifying and Detecting epileptic seizures by analyzing EEG signals. Zuochen Wei proposed a CNN method for automatically detecting epileptic EEG signal segments and seizure events (Craley et al., 2021). These methods have demonstrated excellent performance.

However, the comparative results on the CHB-MIT dataset between the approach and these methods indicate that the designed CNN-based epileptic seizure detection algorithm exhibits superior advancement, stability, and reliability. This is because their CNN algorithms overlooked the importance of different channels in the feature maps during the convolution and pooling processes, leading to a performance loss. The approach incorporated the SE module, which helps reduce this loss by considering the significance of different channels. The SE module explicitly models interdependencies among convolutional feature channels to enhance the network’s representation capability, allowing feature recalibration to be performed (Hu et al., 2020). With this mechanism, global information can selectively emphasize enlightening characteristics and constrain usefulness (Chen et al., 2022). Therefore, introducing the SE module can improve performance metrics such as accuracy (Khan et al., 2022).

Moreover, skip connections were adopted from the FCN architecture, involving the connection of fundamental and advanced attributes. This enables the model better to capture crucial elements at different scales (Liu et al., 2021). Enhancing discriminating epileptic abnormal signals improves classification accuracy, ensuring the model’s robustness and precision. The skip connections facilitate the gradient propagation of shallow features to deeper layers, thereby accelerating the network’s training, learning, and convergence speed (Huang et al., 2022). It helps reduce training time and enhance the training and testing efficacy of the model. Indeed, the CNN model detection algorithm, which combines the SE module with the FCN base, enables more accurate identification of epileptic seizures in different patients. This approach effectively addresses the issue of doctors having to spend significant time and effort reviewing long-term EEG recordings.

### 5.2 Brain area exploration and weighted significance

In addition, the seizure onset zone of epilepsy can be further explored. Dissanayake et al. (2022) proposed two geometric deep learning (GDL) techniques. One approach involved utilizing graphs generated from the physical connections in the electroencephalogram (EEG) grid, while the other employed deep neural network-synthesized diagrams for predicting seizures in epilepsy. This method aids in the localization of epileptic seizures based on scalp EEG (Dissanayake et al., 2022). Narasimhan et al. (2020) utilized non-directional resting-state connectivity measurements to evaluate the connectivity between brain regions in patients and discovered that areas with increased epileptogenicity exhibited higher functional connectivity.

Similarly, the approach identifies seizures in different patients and considers the impact of brain regions on seizure occurrence. The system utilizes the FCN architecture with skip connections and deconvolution (upsampling) method. Deconvolution (upsampling) enlarges the shrunken feature maps to their original dimensions. If upsampling is only applied to the final layer’s feature map to obtain the actual image size, the results are often unsatisfactory. This is because the last layer’s feature map is too small, resulting in the loss of a significant amount of detail. Therefore, global and local effects can be balanced by combining the final layer (which contains global information) with shallower layers (which contain local information) through skip connections.

Visualizing the upsampled feature map of the last layer and normalizing it helps us obtain a weight matrix. In this weight matrix, the value at each position represents the importance of that position for epileptic seizures. By doing this, the classification can be extended from the image-level to the pixel-level. Each row of pixels forms a channel, allowing us to examine each row channel’s weight distribution, thus quantifying each row channel’s contribution to the occurrence of epileptic seizures. Each channel can correspond to specific brain regions using the international 10–20 electrode placement system. This allows us to investigate the significant impact of brain regions such as FZ, CZ, PZ, FT9, FT10, and T8 on the occurrence of epileptic seizures.

### 5.3 Limitations and future work

This paper proposes a compact CNN model on top of the FCN architecture for epileptic seizure detection, integrating the SE module. This algorithm is a method for independent epileptic seizure detection within the same dataset. Nevertheless, building an epileptic seizure detection algorithm that can generalize across different datasets is a more significant challenge. Developing an advanced algorithm for cross-dataset epileptic seizure detection is the next objective, demanding enhanced generalization capabilities from the algorithm.

In addition, while the contribution of EEG channel weights to epileptic seizure detection has been quantified, it is also being explored which brain regions play a significant role in the onset of epileptic seizures. However, the contrast of the weights in the EEG channel map needs to be more evident. Therefore, the algorithm still needs improvement to achieve better visualization and enhance the interpretability of the model.

It has been observed that effective channels are not the same across subjects and they may vary. In order to explore the variation in effective channels and to make the proposed model more generalizable, In the future, a dedicated dataset will be utilized for multicenter cross-validation.

## 6 Conclusion

In this study, a lightweight CNN model incorporating skip connections and deconvolution operations from the FCN architecture was designed. The model was developed explicitly for independent seizure detection in patients. To enhance the model’s ability to recognize seizures, the SE module was introduced, which focuses on channel relationships and automatically helps the model learn the importance of different channel features. The method was evaluated on the CHB-MIT dataset, which consists of long-term continuous EEG data. The approach demonstrated higher G-Mean, ACC, and AUC scores than the baseline methods, indicating superior advancement, stability, and reliability. The contributions of each channel to the seizure detection task have also been quantified, which led us to find that the FZ, CZ, PZ, FT9, FT10, and T8 brain regions have a more pronounced impact on epileptic seizures. This analysis improves the interpretability of the model. In summary, the approach holds great promise in significantly reducing the workload for EEG examinations of epileptic seizures, thus aiding the future clinical application of seizure detection algorithms.

## Data Availability

The original contributions presented in this study are included in this article/Supplementary material, further inquiries can be directed to the corresponding authors.

## References

[B1] Abdallah T. Jrad N. Abdallah F. Humeau-Heurtier A. Van Bogaert P. (2023). A self-attention model for cross-subject seizure detection. *Comput. Biol. Med.* 165:107427. 10.1016/j.compbiomed.2023.107427 37683531

[B2] Abdulwahhab A. H. Abdulaal A. H. Al-Ghrairi A. H. T. Mohammed A. A. Valizadeh M. (2024). Detection of epileptic seizure using EEG signals analysis based on deep learning techniques. *Chaos Solitons Fractals* 181:114700. 10.1016/j.chaos.2024.114700

[B3] Ahmad I. Wang X. Javeed D. Kumar P. Samuel O. W. Chen S. (2023). A hybrid deep learning approach for epileptic seizure detection in EEG SIGNALS. *IEEE J. Biomed. Health Inform.* 10.1109/jbhi.2023.3265983 [Epub ahead of print]. 37037252

[B4] Albaqami H. Hassan G. M. Datta A. (2023). MP-SeizNet: a multi-path CNN Bi-LSTM Network for seizure-type classification using EEG. *Biomed. Signal Process. Control* 84:104780. 10.1016/j.bspc.2023.104780

[B5] Alharbi N. S. Bekiros S. Jahanshahi H. Mou J. Yao Q. J. (2024). Spatiotemporal wavelet-domain neuroimaging of chaotic EEG seizure signals in epilepsy diagnosis and prognosis with the use of graph convolutional LSTM networks. *Chaos Solitons Fractals* 181:114675. 10.1016/j.chaos.2024.114675

[B6] Chakrabarti S. Swetapadma A. Pattnaik P. K. (2021). A channel independent generalized seizure detection method for pediatric epileptic seizures. *Comput. Methods Programs Biomed.* 209:106335. 10.1016/j.cmpb.2021.106335 34390934

[B7] Chen G. Yin J. Dai Y. Zhang J. Yin X. Cui L. (2022). A novel convolutional neural network for kidney ultrasound images segmentation. *Comput. Methods Programs Biomed.* 218:106712. 10.1016/j.cmpb.2022.106712 35248816

[B8] Chen Y. B. Mancini M. Zhu X. T. Akata Z. (2024). Semi-supervised and unsupervised deep visual learning: a survey. *IEEE Trans. Pattern Anal. Mach. Intellig.* 46 1327–1347. 10.1109/tpami.2022.3201576 36006881

[B9] Chou C. H. Shen T. W. Tung H. Hsieh P. F. Kuo C. E. Chen T. M. (2023). Convolutional neural network-based fast seizure detection from video electroencephalograms. *Biomed. Signal Process. Control* 80:104380. 10.1016/j.bspc.2022.104380

[B10] Craley J. Johnson E. Jouny C. Venkataraman A. (2021). Automated inter-patient seizure detection using multichannel Convolutional and Recurrent Neural Networks. *Biomed. Signal Process. Control* 64:102360. 10.1016/j.bspc.2020.102360

[B11] Dissanayake T. Fernando T. Denman S. Sridharan S. Fookes C. (2022). Geometric deep learning for subject independent epileptic seizure prediction using scalp EEG signals. *IEEE J. Biomed. Health Inform.* 26 527–538. 10.1109/jbhi.2021.3100297 34314363

[B12] Du Y. P. Liu J. (2022). IENet: a robust convolutional neural network for EEG based brain-computer interfaces. *J. Neural Eng.* 19 036031. 10.1088/1741-2552/ac7257 35605585

[B13] Duan L. J. Wang Z. Y. Qiao Y. H. Wang Y. Huang Z. Y. Zhang B. C. (2022). An automatic method for epileptic seizure detection based on deep metric learning. *IEEE J. Biomed. Health Inform.* 26 2147–2157. 10.1109/jbhi.2021.3138852 34962890

[B14] Eris F. G. Akbal E. (2024). Enhancing speech emotion recognition through deep learning and handcrafted feature fusion. *Appl. Acoust.* 222:110070. 10.1016/j.apacoust.2024.110070

[B15] Fiest K. M. Sauro K. M. Wiebe S. Patten S. B. Kwon C. S. Dykeman J. (2017). Prevalence and incidence of epilepsy: a systematic review and meta-analysis of international studies. *Neurology* 88 296–303. 10.1212/wnl.0000000000003509 27986877 PMC5272794

[B16] Hu D. H. Cao J. W. Lai X. P. Wang Y. M. Wang S. Ding Y. (2021). Epileptic state classification by fusing hand-crafted and deep learning EEG features. *IEEE Trans. Circ. Syst. II Express Briefs* 68 1542–1546. 10.1109/tcsii.2020.3031399

[B17] Hu J. Shen L. Albanie S. Sun G. Wu E. (2020). Squeeze-and-excitation networks. *IEEE Trans. Pattern Anal. Mach. Intell.* 42 2011–2023. 10.1109/tpami.2019.2913372 31034408

[B18] Hu S. C. Liu J. Yang R. Wang Y. N. Wang A. G. Li K. Z. (2023). Exploring the applicability of transfer learning and feature engineering in epilepsy prediction using hybrid transformer model. *IEEE Trans. Neural Syst. Rehabil. Eng.* 31 1321–1332. 10.1109/tnsre.2023.3244045 37027542

[B19] Huang G. Liu Z. Pleiss G. Maaten L. V. Weinberger K. Q. (2022). Convolutional networks with dense connectivity. *IEEE Trans. Pattern Anal. Mach. Intell.* 44 8704–8716. 10.1109/tpami.2019.2918284 31135351

[B20] Jaiswal A. Nenonen J. Stenroos M. Gramfort A. Dalal S. S. Westner B. U. (2020). Comparison of beamformer implementations for MEG source localization. *Neuroimage* 216:116797. 10.1016/j.neuroimage.2020.116797 32278091 PMC7322560

[B21] Jiang Y. A. Li Y. Zou S. R. Zhang H. K. Bai Y. P. (2021). Hyperspectral image classification with spatial consistence using fully convolutional spatial propagation network. *IEEE Trans. Geosci. Remote Sens.* 59 10425–10437. 10.1109/tgrs.2021.3049282

[B22] Kaur A. Puri V. Shashvat K. Maurya A. K. (2022). Automated identification of inter-ictal discharges using residual deep learning neural network amidst of various artefacts. *Chaos Solitons Fractals* 156:111886. 10.1016/j.chaos.2022.111886

[B23] Khan F. A. Umar Z. Jolfaei A. Tariq M. (2024). Explainable fuzzy deep learning for prediction of epileptic seizures using EEG. *IEEE Trans. Fuzzy Syst.* 32 5428–5437. 10.1109/tfuzz.2024.3434709

[B24] Khan M. M. Uddin M. S. Parvez M. Z. Nahar L. (2022). A squeeze and excitation ResNeXt-based deep learning model for Bangla handwritten compound character recognition. *J. King Saud Univ. Comput. Inform. Sci.* 34 3356–3364. 10.1016/j.jksuci.2021.01.021

[B25] Lawhern V. J. Solon A. J. Waytowich N. R. Gordon S. M. Hung C. P. Lance B. J. (2018). EEGNet: a compact convolutional neural network for EEG-based brain-computer interfaces. *J. Neural Eng.* 15:056013. 10.1088/1741-2552/aace8c 29932424

[B26] LeCun Y. Bengio Y. Hinton G. (2015). Deep learning. *Nature* 521 436–444. 10.1038/nature14539 26017442

[B27] Li C. Huang X. Y. Song R. C. Qian R. B. Liu X. Chen X. (2022). EEG-based seizure prediction via transformer guided CNN. *Measurement* 203:111948. 10.1016/j.measurement.2022.111948

[B28] Liang W. X. Pei H. J. Cai Q. L. Wang Y. H. (2020). Scalp EEG epileptogenic zone recognition and localization based on long-term recurrent convolutional network. *Neurocomputing* 396 569–576. 10.1016/j.neucom.2018.10.108

[B29] Liu G. Tian L. Zhou W. (2022). Patient-independent seizure detection based on channel-perturbation convolutional neural network and bidirectional long short-term memory. *Int. J. Neural Syst.* 32:2150051. 10.1142/s0129065721500519 34781854

[B30] Liu Q. Wu Z. B. Jia X. P. Xu Y. Wei Z. H. (2021). From local to global: class feature fused fully convolutional network for hyperspectral image classification. *Remote Sens.* 13:5043. 10.3390/rs13245043

[B31] Liu X. L. Richardson A. G. (2021). Edge deep learning for neural implants: a case study of seizure detection and prediction. *J. Neural Eng.* 18:046034. 10.1088/1741-2552/abf473 33794507

[B32] Luo S. W. Ivison H. Han S. C. Poon J. (2024). Local interpretations for explainable natural language processing: a survey. *ACM Comput. Surv.* 56:232. 10.1145/3649450

[B33] Narasimhan S. Kundassery K. B. Gupta K. Johnson G. W. Wills K. E. Goodale S. E. (2020). Seizure-onset regions demonstrate high inward directed connectivity during resting-state: an SEEG study in focal epilepsy. *Epilepsia* 61 2534–2544. 10.1111/epi.16686 32944945 PMC7899016

[B34] Nasiri S. Clifford G. D. (2021). Generalizable seizure detection model using generating transferable adversarial features. *IEEE Signal Process. Lett.* 28 568–572. 10.1109/lsp.2021.3060967

[B35] O’Shea A. Lightbody G. Boylan G. Temko A. (2020). Neonatal seizure detection from raw multi-channel EEG using a fully convolutional architecture. *Neural Netw.* 123 12–25. 10.1016/j.neunet.2019.11.023 31821947

[B36] Ozdemir M. A. Cura O. K. Akan A. (2021). Epileptic EEG classification by using time-frequency images for deep learning. *Int. J. Neural Syst.* 31:2150026. 10.1142/s012906572150026x 34039254

[B37] Park Y. S. Cosgrove G. R. Madsen J. R. Eskandar E. N. Hochberg L. R. Cash S. S. (2020). Early detection of human epileptic seizures based on intracortical microelectrode array signals. *IEEE Trans. Biomed. Eng.* 67 817–831. 10.1109/tbme.2019.2921448 31180831 PMC7067044

[B38] Qiu S. Joshi P. S. Miller M. I. Xue C. Zhou X. Karjadi C. (2020). Development and validation of an interpretable deep learning framework for Alzheimer’s disease classification. *Brain* 143 1920–1933. 10.1093/brain/awaa137 32357201 PMC7296847

[B39] Rasheed K. Qadir J. O’Brien T. J. Kuhlmann L. Razi A. (2021). A generative model to synthesize EEG data for epileptic seizure prediction. *IEEE Trans. Neural Syst. Rehabil. Eng.* 29 2322–2332. 10.1109/tnsre.2021.3125023 34727036 PMC8592500

[B40] Shelhamer E. Long J. Darrell T. (2017). Fully convolutional networks for semantic segmentation. *IEEE Trans. Pattern Anal. Mach. Intell.* 39 640–651. 10.1109/tpami.2016.2572683 27244717

[B41] Shen Y. Zhu S. J. Chen C. Du Q. Xiao L. Chen J. Y. (2021). Efficient deep learning of nonlocal features for hyperspectral image classification. *IEEE Trans. Geosci. Remote Sens.* 59 6029–6043. 10.1109/tgrs.2020.3014286

[B42] Si X. P. Yang Z. B. Zhang X. J. Sun Y. L. Jin W. P. Wang L. (2023). Patient-independent seizure detection based on long-term iEEG and a novel lightweight CNN. *J. Neural Eng.* 20:016037. 10.1088/1741-2552/acb1d9 36626831

[B43] Si X. Zhang X. Zhou Y. Chao Y. Lim S. N. Sun Y. (2021). White matter structural connectivity as a biomarker for detecting juvenile myoclonic epilepsy by transferred deep convolutional neural networks with varying transfer rates. *J. Neural Eng.* 18 10.1088/1741-2552/ac25d8 34507303

[B44] Singh K. Malhotra J. (2022). Two-layer LSTM network-based prediction of epileptic seizures using EEG spectral features. *Complex Intellig. Syst.* 8 2405–2418. 10.1007/s40747-021-00627-z

[B45] Staba R. J. Stead M. Worrell G. A. (2014). Electrophysiological biomarkers of epilepsy. *Neurotherapeutics* 11 334–346. 10.1007/s13311-014-0259-0 24519238 PMC3996122

[B46] Tuncer E. Bolat E. D. (2022). Classification of epileptic seizures from electroencephalogram (EEG) data using bidirectional short-term memory (Bi-LSTM) network architecture. *Biomed. Signal Process. Control* 73:103462. 10.1016/j.bspc.2021.103462

[B47] Xia Y. Zhou W. Li C. Yuan Q. Geng S. (2015). Seizure detection approach using S-transform and singular value decomposition. *Epilepsy Behav.* 52(Pt. A) 187–193. 10.1016/j.yebeh.2015.07.043 26439656

[B48] Xu G. Ren T. Chen Y. Che W. (2020). A one-dimensional CNN-LSTM model for epileptic seizure recognition using EEG signal analysis. *Front. Neurosci.* 14:578126. 10.3389/fnins.2020.578126 33390878 PMC7772824

[B49] Zhang X. Yao L. Dong M. Liu Z. Zhang Y. Li Y. (2020). Adversarial representation learning for robust patient-independent epileptic seizure detection. *IEEE J. Biomed. Health Inform.* 24 2852–2859. 10.1109/jbhi.2020.2971610 32071011

