# Supplementary material for: Application of deconvolutional networks for feature interpretability in epilepsy detection
Source: Front Neurosci. 2025 Jan 24;18:1539580. doi: 10.3389/fnins.2024.1539580 (PMC11802560; doi:10.3389/fnins.2024.1539580)
Supplement: Supplementary file 1 [file Data_Sheet_1.docx]

**Supplementary Materials**

Sihao Shao

December 28, 2024

Based on the analysis of the weighting situation of each channel at the time of the first seizure in patient chb04 in the CHBMIT dataset, the situation was further analyzed for all patients. Below is a graph of the weighting channels of the SE model of this paper at the time of the first seizure of the 23 patients in the dataset.


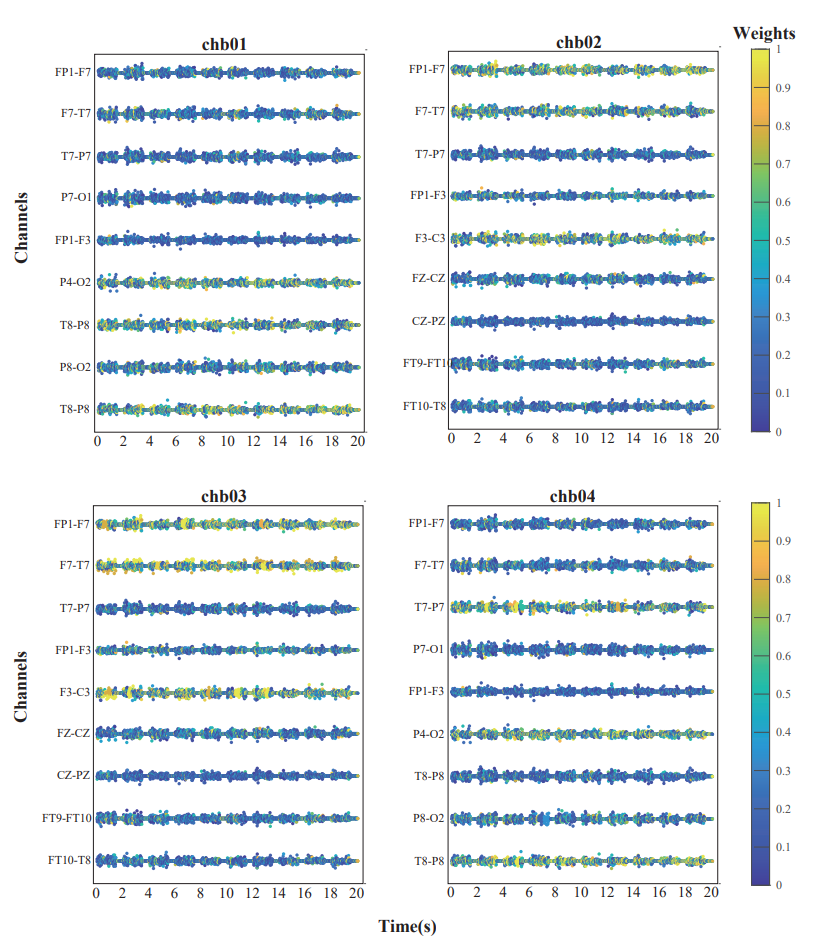


For chb01 patients, the channel weights corresponding to the brain regions P4-O2,T8-P8 were larger; for chb02 and chb03 patients, the channel weights corresponding to the brain regions FP1-F7, F7-T7, and F3-C3 were larger; and for chb04 patients, the channel weights corresponding to the brain regions T7-P7, P4-O2, and T8-P8 were larger;


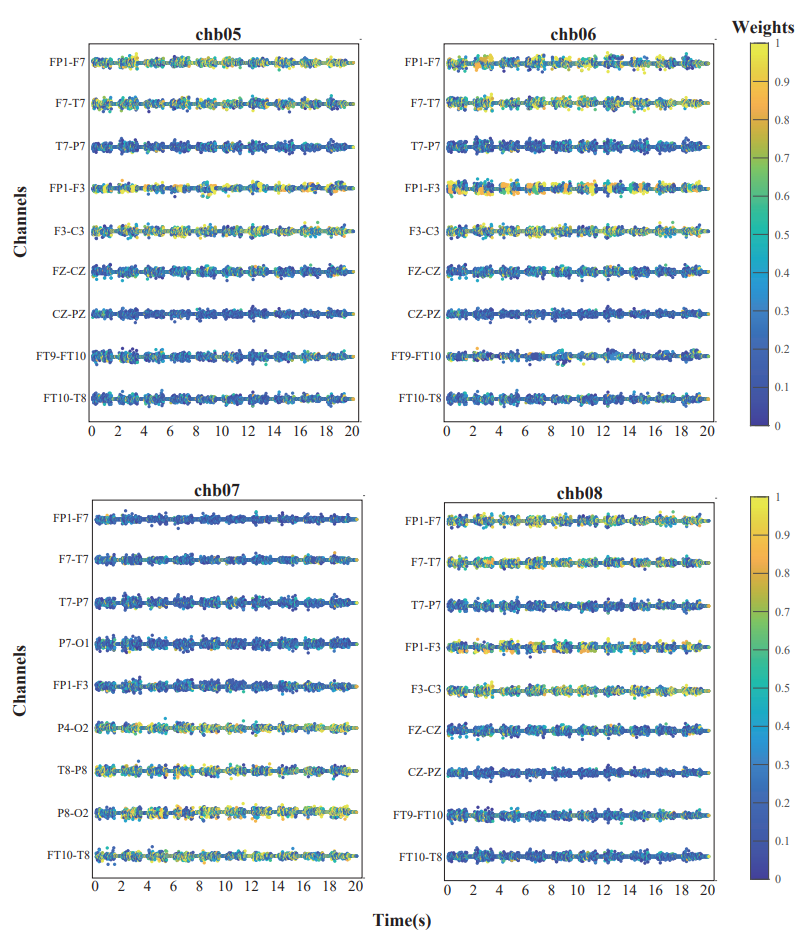


For chb05, chb06, and chb08 patients, the brain regions FP1-F7, F7-T7, FP1-F3, and F3-C3 corresponded to larger channel weights; for chb07 patients, the brain regions P2-O2, T8-P8, P8-O2, and FT10-T8 corresponded to larger channel weights;


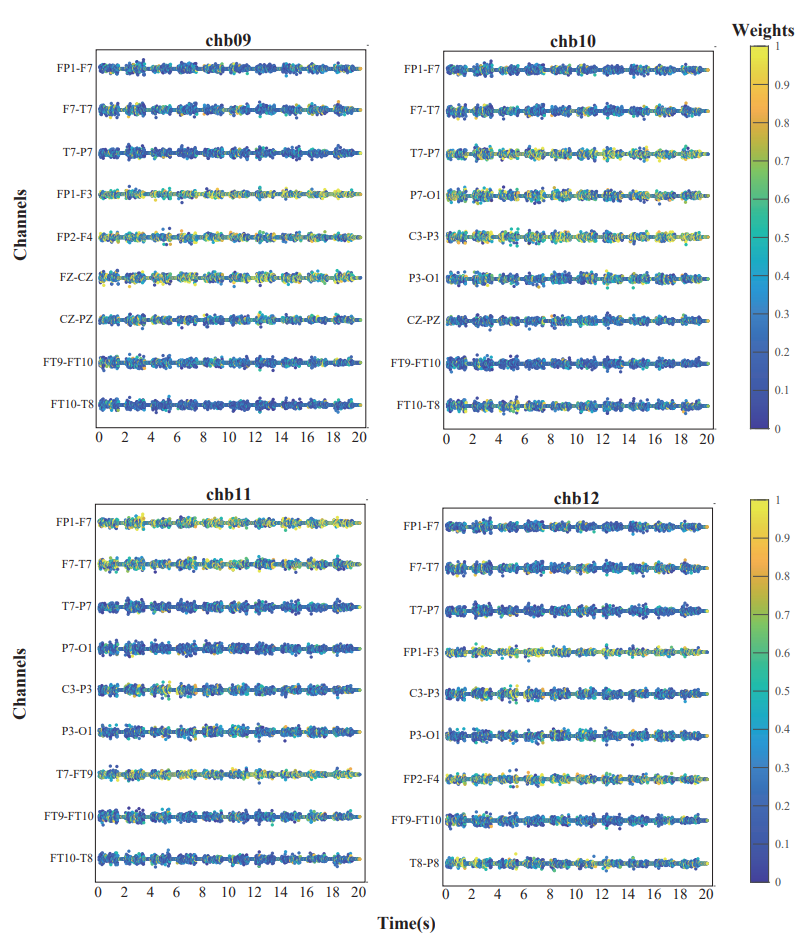


For chb09 patients, the brain regions FP1-F3, FP2-F4, and FZ-CZ corresponded to larger channel weights; for chb10 patients, the brain regions T7-P7, P7-O1, and C3-P3 corresponded to larger channel weights;


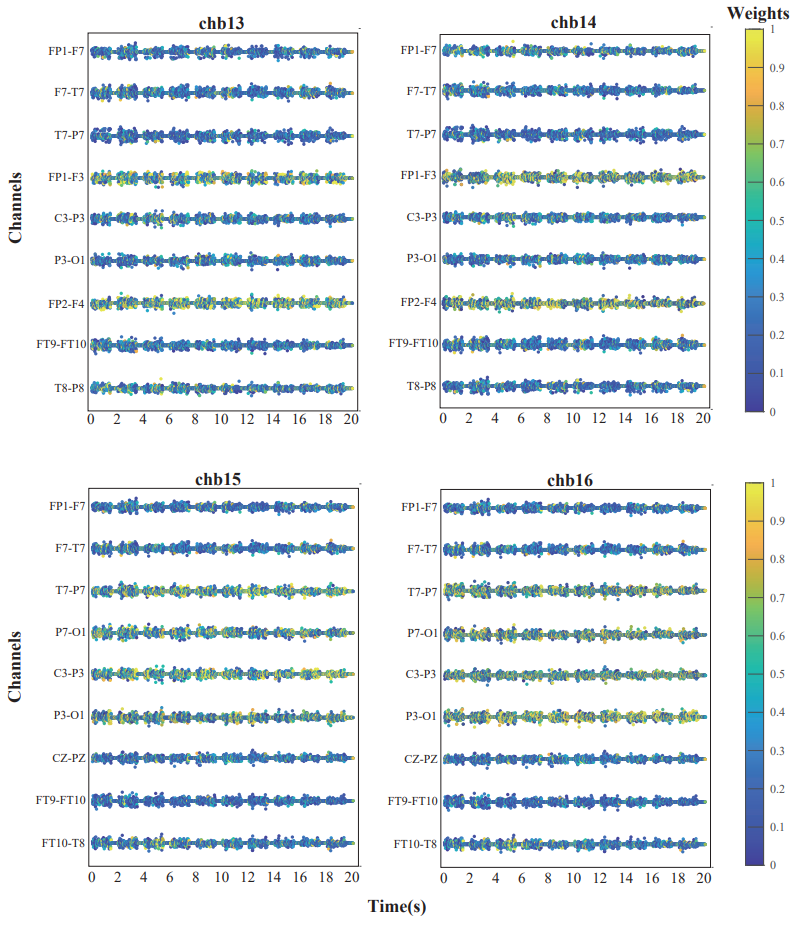


For chb11, chb19, chb20, chb21, and chb22 patients, the brain regions FP1-F7, F7-T7, and T7-FT9 corresponded to larger channel weights; for chb12, chb13, and chb14 patients, the brain regions FP1-F3 and FP2-F4 corresponded to larger channel weights;


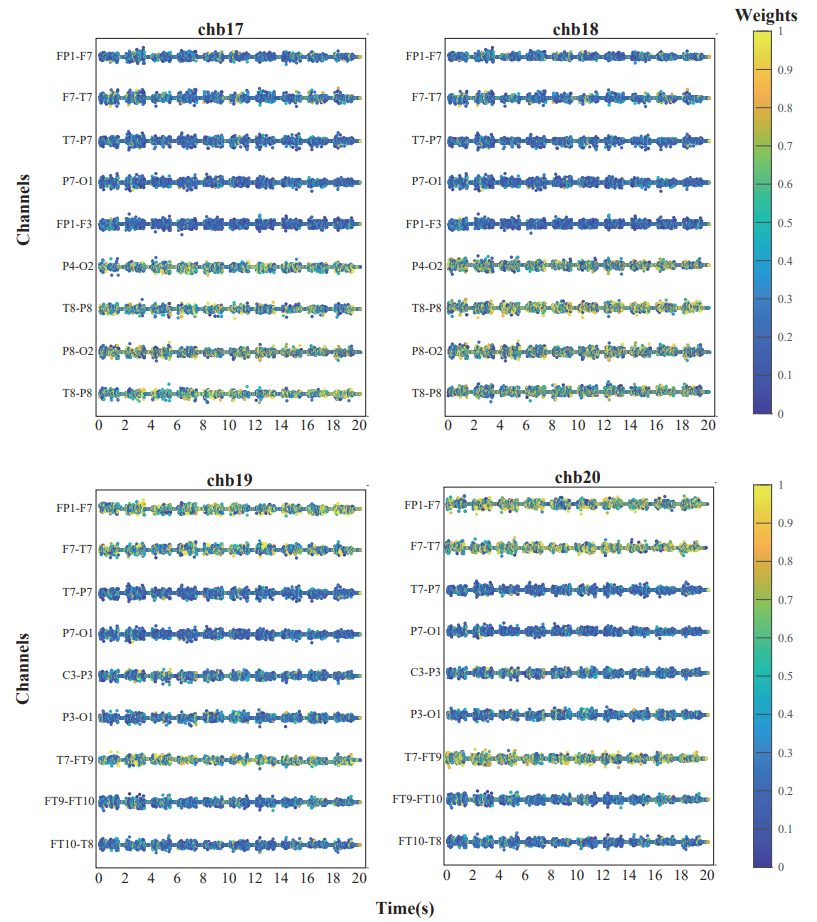


For chb15 and chb16 patients, T7-p7, P7-O1, C3-P3, P3-O1 these brain regions corresponded to larger channel weights; for chb17 and chb18 patients, P4-O2, T8-P8, P8-O2 these brain regions corresponded to larger channel weights; for chb23 patients, FP1-F7, FP1-F3,F3- C3 these brain regions corresponded to channels with larger weights.


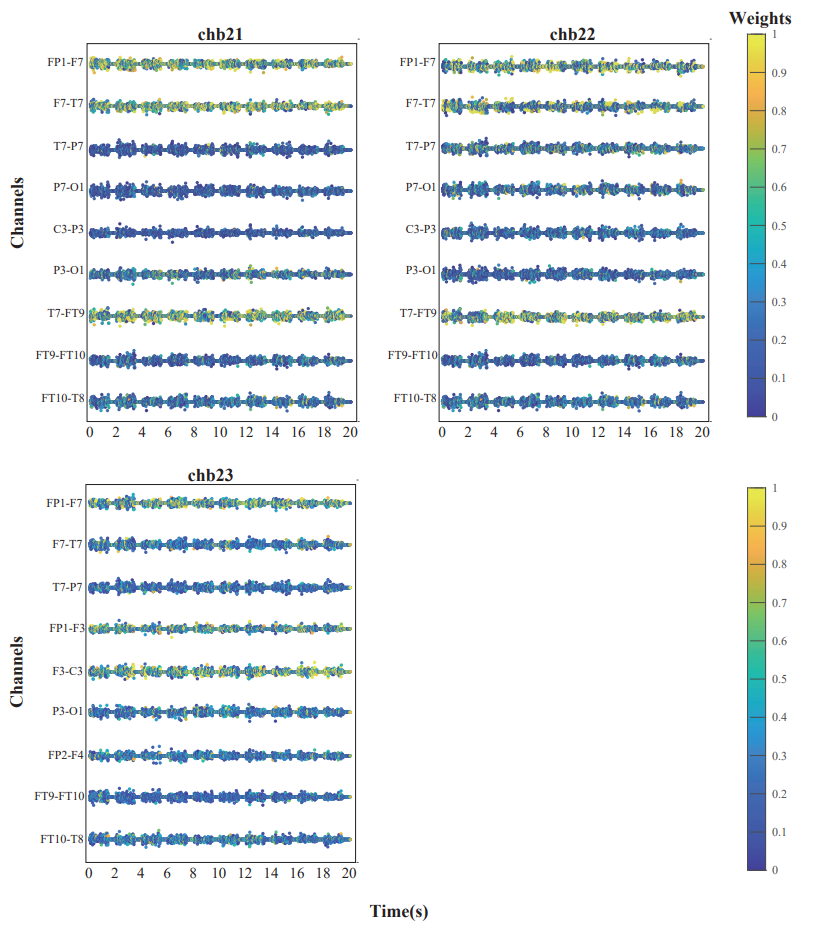


By visualizing the EEG feature maps of all patients in the CHBMIT dataset, the weights of epileptogenic channels were derived separately for each patient, which were corresponded to brain regions by the international 10-20 lead system to explore which brain regions play a significant role in epileptogenesis in each patient. The interpretability of the model was further enhanced.
